# Knowledge on Alzheimer's Disease among Public Hospitals and Health Clinics Pharmacists in the State of Selangor, Malaysia

**DOI:** 10.3389/fphar.2017.00739

**Published:** 2017-10-26

**Authors:** Thimarul Huda Mat Nuri, Yet Hoi Hong, Long Chiau Ming, Suhaidah Mohd Joffry, Muhamad Faiz Othman, Chin Fen Neoh

**Affiliations:** ^1^Department of Pharmacy Practice, Faculty of Pharmacy, Universiti Teknologi MARA, Puncak Alam, Malaysia; ^2^Department of Physiology, Faculty of Medicine, University of Malaya, Kuala Lumpur, Malaysia; ^3^School of Pharmacy, KPJ Healthcare University College, Nilai, Malaysia; ^4^Unit for Medication Outcomes Research and Education, Pharmacy, School of Medicine, University of Tasmania, Hobart, TAS, Australia; ^5^Vector-Borne Diseases Research Group, Pharmaceutical and Life Sciences Community of Research, Universiti Teknologi MARA, Puncak Alam, Malaysia; ^6^Collaborative Drug Discovery Research Group, Pharmaceutical and Life Sciences Community of Research, Universiti Teknologi MARA, Shah Alam, Malaysia

**Keywords:** Neurodegenerative diseases, dementia, Alzheimer's disease knowledge scale

## Abstract

The prevalence of Alzheimer's disease (AD) has increased with the fast growing of aging population, thereby posing great challenges to provision of care for AD patients. Pharmacists play a vital role in the management of AD; this includes recognizing early symptoms of AD, providing medication counseling to AD patients and their caretakers, and identifying potential adverse drug reactions. A comprehensive understanding of the disease progression, as well as the pharmacological therapy, is essential to provide effective care to AD patients. The level of knowledge about AD among the pharmacists, however, remains unknown. Hence, this study aimed to assess the knowledge on AD among the pharmacists in public hospitals and health clinics and its correlates. A clear picture of the characteristics associated with different levels of knowledge could facilitate the targeted re-training of pharmacists. The 30-item validated Alzheimer disease knowledge scale (ADKS) tool was pilot-tested and used in this cross-sectional study. All pharmacists, from nine public hospitals and seven public health clinics in the State of Selangor, Malaysia, were invited to participate in this cross-sectional survey. The ADKS score was computed and compared across demographics characteristics. A total of 445 pharmacists responded to the survey. These pharmacists had a moderate overall score in ADKS; nevertheless, high scores were recorded in the domains of treatment management and care giving. No difference in AD knowledge was found among pharmacists worked in public hospitals and health clinics, except for the domain of care giving (*p* = 0.033). Ethnicity and age group were independent predictors of ADKS score in the current study. The pharmacists in the current study had moderate AD knowledge. On-going education and training programme on AD, in particular the domains other than treatment management and care giving, should be provided to the pharmacists to ensure delivery of quality care to AD patients.

## Introduction

More than 46 million people worldwide are living with Alzheimer's disease (AD) and the number is expected to increase to 131.5 million by 2050, with the majority are from low and middle income countries (Alzheimer's Disease International, 2015). The estimated cost of managing AD was at US$818 billion and it is forecasted to increase to a trillion dollar by 2018 (Alzheimer's Disease International, 2015). The prevalence of dementia in Malaysia is estimated at 0.126% and 0.454% in 2020 and 2050, respectively (Rees et al., 2006; Tey et al., 2016). This is due to the fact that most family members view AD's symptoms as normal aging, and hence, do not seek for any medical treatments (Alzheimer's Disease International, 2015).

Management of AD patients is complex as the development of psychiatric and behavioral disturbances upon pharmacological treatment might overlap with the symptoms of cognitive decline (Feinberg and Michocki, 1998). Pharmacists are a valuable member of the healthcare team in managing patients with AD. They are trained to identify potential adverse drug reactions (ADR), helping the clinicians to rule out the possibility of these ADRs that may appear to be AD symptoms (Marasco et al., 2003). In addition, pharmacists can monitor for the safety and efficacy of medications and medication adherence among the AD patients (Chang et al., 2015). Pharmacists can educate AD patients and their caregivers, ensuring that they keep abreast with new drug therapies and updated resources (Chang et al., 2015). More recently, the role of pharmacists in the early detection of AD is increasingly emphasized as they are highly visible and easily accessed by the community (Criddle, 2014).

Inadequate knowledge in regards to AD among the healthcare professionals have been shown to be negatively impacted patient outcomes (Barrett et al., 1997; Perry et al., 2008). Studies have revealed that some primary care physicians failed to properly evaluate and manage patients with AD due to inadequate training (Barrett et al., 1997). A recent survey conducted among community pharmacists in Maltese Islands revealed that the respondents generally had inadequate knowledge on risk factors, caregiving issues, and pharmacotherapy of AD (Zerafa and Scerri, 2016). These indicate a need for continuous knowledge empowerment on AD among the healthcare professionals in order to provide better care to AD patients (Rubio-Valera et al., 2014). Limited studies have been conducted to assess the knowledge on AD among the healthcare professionals (Barrett et al., 1997; Smyth et al., 2013), in particular the pharmacists in the public healthcare institutions whom are regularly accessed by the public. Accordingly, this study aimed to (i) assess the AD knowledge among the pharmacists working in the public hospitals and health clinics in the state of Selangor, Malaysia, and (ii) correlate the demographics that affect pharmacists' knowledge on AD.

## Materials and methods

### Participants and settings

This cross-sectional study was conducted between September and November 2016. All pharmacists who worked in public hospitals and health clinics in Selangor, Malaysia, were invited to participate in this study. The list of the pharmacists with their email addresses was obtained from Selangor State Health Department.

### Questionnaire and data collection

A 38-item survey, which included questions on: (i) demographics of the pharmacists, (ii) validated items from Alzheimer's disease Knowledge Scale (ADKS) (Carpenter et al., 2009), and (iii) self-rated AD knowledge level, was used in this study. This instrument was selected to measure the knowledge of AD due to its ease of use, demonstrated reliability and validity, and also its suitability to be applied for diverse pools of respondents including general public, caregivers, and healthcare professionals (Smyth et al., 2013). The ADKS consisted of 30 true/false items, which further divided into seven content domains (i.e., risk factors, assessment and diagnosis, symptoms, course, life impact, caregiving, and treatment and management). The total ADKS score was calculated by adding the correct responses to produce a score that ranges from 0 to 30. Moreover, the respondents were asked to self-rate their level of knowledge on AD based on a scale from 0 = I know nothing at all to 10 = I am very knowledgeable. This survey took about 10–15 min to complete. A pilot study was conducted among 33 pharmacists (which were not included into the data analysis) to determine face and content validity as well as reliability of ADKS. The Cronbach's alpha value was 0.6, indicating an internal consistency of ADKS among this cohort (Bhatnagar et al., 2014).

The survey was distributed via two approaches: (i) using a web-based questionnaire, hosted by www.surveymonkey.com (for those pharmacists who worked in hospitals and health clinics at 15 km or more from the city center) and (ii) manual distribution of hard copy of the survey (for those pharmacists who worked in nearby hospitals and health clinics). For web-based survey, an invitation email containing a detailed explanatory statement for participation and a link to the survey were sent to all pharmacists. The first reminder was sent 2 weeks after the initial invitation email and a total of three reminders were sent within the survey period. For the manually distributed survey, the surveys were collected 2 weeks after the distribution.

### Ethical approval

Ethics approvals from Research Ethics Committee (REC) of Universiti Teknologi MARA (UiTM) [Reference no: 600-IRMI (5/1/6) REC/345/16] and Medical Research and Ethics Committee (MREC) [Reference no: NMRR-15-133-24229(IIR)], Ministry of Health, Malaysia, were obtained for the purpose of this study.

### Statistical analyses

Descriptive data were analyzed by using Statistical Package for Social Science (SPSS) programme version 20.0 and Microsoft Excel version 2010. Descriptive statistical analysis, such as frequency, percentage, mean, and standard deviation (SD) was carried out to analyze demographic data. Descriptive statistics for all independent variables were calculated and the mean ADKS scores across different groups were compared using independent *t*-tests and ANOVA test. The predictor of ADKS score was determined using multiple linear regression.

## Results

### Demographics characteristics

Of the total 775 pharmacists, 445 responded to the survey, giving a response rate of 57.4%. There was almost an equal participation of pharmacists from the public hospital and health clinics (Table 1). The demographics of the respondents are shown in Table 1. The respondents were mainly female (83.4%). Half of the respondents were Malay (53.5%), followed by Chinese (35.5%), Indian (10.3%), and other ethnicities (0.7%). The mean age of the respondents was 28.94 ± 3.37 years old. The majority of the respondents had Bachelor degree (92.8%) and the reminders were Master holders. Most of them have working experience of 4–7 years (42.2%) and only 2.2% of the respondents have worked for 12–14 years. The respondents were mainly from outpatient pharmacy (57.1%), followed by inpatients pharmacy (12.1%). Only 1.6% was from the parenteral nutrition unit.

**Table 1:** Demographic characteristics and their relationship to AD knowledge (*n* = 445).

**Demographic characteristics**	**n (%)**	**Mean ADKS score (SD)**	***F***	***df***	***P*-value**
**GENDER**					
Male	74 (16.6)	19.23 (4.17)	1.054	443	0.225^a^
Female	371 (83.4)	18.67 (3.52)			
**EDUCATION LEVEL**					
Bachelor degree	414 (92.8)	18.83 (3.66)	0.2	443	0.655^a^
Master degree	31 (7.0)	17.90 (3.36)			
**PREMISES**					
Health clinic	225 (50.6)	19.05 (3.69)	0.142	443	0.095^a^
Hospital	220 (49.4)	18.47 (3.56)			
**AGE GROUP**					
<30	335 (75.3)	19.03 (3.69)	3.43	2,442	0.009^b^^,^^c^
31–40	106 (23.8)	17.99 (3.44)			
41–50	4 (0.9)	18.06 (1.03)			
**ETHNICITY**					
Malay	238 (53.5)	19.21 (3.86)	2.82	3,441	0.039^b^^,^^c^
Chinese	158 (35.5)	18.13 (3.24)			
Indian	46 (10.3)	18.72 (3.64)			
Others	3 (0.7)	18.33 (0.58)			
**WORKING EXPERIENCE**					
<3	174 (39.1)	18.73 (3.68)	0.172	4,440	0.953^b^
4–7	188 (42.2)	18.80 (3.61)			
8–11	70 (15.7)	18.65 (3.58)			
12–14	10 (2.2)	19.00 (6.16)			
>15	3 (0.7)	18.76 (3.64)			
**PHARMACY UNIT**					
Outpatient pharmacy	254 (57.1)	19.21 (3.76)	2.299	9,435	0.016^b^^,^^c^
Store	47 (10.6)	17.55 (2.67)			
Clinical pharmacy	54 (12.1)	17.81 (3.11)			
Drug information services	23 (5.2)	17.91 (3.78)			
Total parenteral nutrition	7 (1.6)	21.14 (4.05)			
Others	60 (13.4)	17.16 (3.05)			

a *Independent t-test*.

b *One-way ANOVA test (post-hoc test LSD procedure)*.

c *P-value is significant (< 0.05)*.

### Relationships between AD knowledge and demographic characteristics

The mean (SD) total ADKS score for health clinics pharmacists was 19.05 (3.69), with 63.5% correct answers, whereas hospital pharmacists' score was 18.47 (3.56), with 62.5% correctness (Table 1). Both scores were categorized as intermediate score with no significant difference detected between the pharmacists from public hospitals and health clinics (*p* = 0.095). Significant differences in total ADKS score were noted for different age group (*p* = 0.009), ethnicity (*p* = 0.039), and pharmacy unit (*p* = 0.016) of the respondents (Table 1). Interestingly, ADKS score for this cohort was independent of the education level [i.e., Master vs. Bachelor (*p* = 0.655)]. As for self-rated AD knowledge, the respondents rated themselves as having average AD knowledge, with a mean (SD) of 4.22 (1.77), based on a 11-point scale. There was a significant correlation between self-rated knowledge and ADKS score, whereby the respondents who rated low knowledge tended to have lower ADKS score (*r* = 0.33, *p* = 0.001).

### Difference in ADKS domains scores between public hospitals and health clinics pharmacists

As shown in Table 2, pharmacists from both public hospitals and health clinics had lower percentage of correctness in two of the domains, namely symptoms (46.2%) and course of disease (48.3%). On the other hand, all pharmacists had higher percentage of correctness in the domain of treatment management and care giving, with a score of 76.4 and 72.1%, respectively. For the domain of life impact (52.7%) and risk factors (59.8%), all pharmacists in this study had average percentage of correctness. There was no significant difference in the mean scores between the public hospital and health clinics pharmacists in any of the domain except the domain of care giving where the public hospital pharmacists scored significantly higher than the health clinics pharmacists (*p* = 0.033).

**Table 2:** ADKS content domain scores between public hospitals and health clinics pharmacists.

**Domain**	**No of Item**	**Mean (SD) ADKS score for both groups**	**% correct**	**Mean (SD) ADKS score**		***P*-value^a^**
				**Hospital pharmacists (*n* = 220)**	**Health clinics pharmacists (*n* = 225)**	
Life impact	3	1.58 (0.77)	52.7	1.58 (0.53)	1.57 (0.05)	0.898
Risk factors	6	3.59 (1.22)	59.8	3.64 (0.08)	3.53 (0.82)	0.356
Symptoms	4	1.84 (1.05)	46.2	1.92 (0.07)	1.77 (0.07)	0.131
Treatment management	4	3.05 (0.81)	76.4	3.06 (0.05)	3.04 (0.05)	0.829
Assessment	4	2.67 (0.91)	66.8	2.67 (0.06)	2.66 (0.58)	0.932
Care giving	5	3.60 (1.07)	72.1	3.71 (0.06)	3.49 (0.07)	0.033^b^
Course of disease	4	2.41 (1.00)	48.3	2.44 (0.66)	2.38 (0.068)	0.437

a *Independent t-test*.

b *P value is significant (< 0.05)*.

### Predictors of ADKS score

Findings from multiple linear regression analysis (Table 3) revealed that aged <30 and Malay ethnicity as independent predictors of ADKS score. Malay ethnicity had 0.9 point higher ADKS score compared to Chinese (adjusted *B* = 0.89, 95% *CI* = 0.219, 1.557, *p* = 0.009). Those respondents aged <30 had 0.8 point higher ADKS score compared those aged between 31 and 40 (adjusted *B* = 0.83, 95% *CI* = 0.059, 1.600, *p* = 0.018).

**Table 3:** Regression model predicting knowledge of AD among pharmacists.

**Variables**	**Single linear regression**		**Multiple linear regression**	
	**B^a^ (95% CI)**	***P*-value**	**B^b^ (95% CI)**	***P*-value**
**PREMISES**				
Health clinic	–	–	–	–
Hospital	−0.54(−1.220, 0.135)	0.116		
**GENDER**				
Male	–	–		
Female	−0.56(1.473, 0.347)	0.225		
**EDUCATION LEVEL**				
Bachelor degree	–	–		
Master degree	−0.93(−2.261, 0.401)	0.170		
**AGE GROUP**				
<30	1.035 (0.285, 1.811)	0.009^c^	0.83 (0.059, 1.600)	0.018^c^
31−40	–			
41−50	0.75 (−2.87, 4.371)	0.684		
**ETHNICITY**				
Chinese	–	–		
Malay	1.078 (0.348, 1.809)	0.004^c^	0.89 (0.219, 0.557)	0.009^c^
India	0.598 (−0.602, 1.78)	0.331		
Others	0.206 (−3.939, 4.351)	0.922		

a *Crude regression coefficient*.

b *Adjusted regression coefficient*.

c *P-value is significant (< 0.05)*.

*Forward multiple linear regression method applied. Model assumption are fulfilled. There was no interaction amongst independent variable. No multicollinearity detected. Coefficient of determination (R^2^) = 0.044*.

## Discussion

The role of pharmacists in management of AD is increasingly recognized, which includes recognizing early symptoms of AD, referring AD patients to seek for timely diagnosis and treatment, identifying potential ADRs of pharmacological treatment, and educating and providing counseling to the AD patients and caregivers. For example, findings from a study on multi-disciplinary dementia care services conducted in Germany revealed that pharmacists are adapt in identifying problems related to drug administration, adherence, and drug interaction among patients with dementia (Wucherer et al., 2017). Meanwhile, in the United Kingdom, a trial targeting people with dementia initiated on anti-psychotics demonstrated that pharmacist-led medication review successfully limited the prescribing of anti-psychotics to people with dementia because of the increased risk of ADRs (Child et al., 2012). In Japan, study involving hospital pharmacists on donepezil consultation for patients with AD and their caregivers has demonstrated an enhanced medication adherence though this drug could cause insomnia and gastrointestinal disturbance (Watanabe et al., 2012). In Malaysia, while medication reviews for patients with other chronic diseases have been implemented by hospital pharmacists via medication therapy adherence clinics (Lim et al., 2016; Aidit et al., 2017), this has not been done for AD patients. It is important to ensure that the pharmacists are equipped with proficient knowledge on AD because poor management in AD can result in side effects, inappropriate dosing, and non-compliance to medications (Stafford, 2015).

This study, the first in Asia region, assessed the level of AD knowledge among the pharmacists in public hospitals and health clinics by using ADKS. This study revealed that the pharmacists from both public hospitals and health clinics in the state of Selangor had moderate knowledge on AD. It is unknown whether the current scores are on par with the regional and international counterparts as no similar study was conducted except one involving community pharmacists at Maltese Islands (Zerafa and Scerri, 2016). The score attained by the Malaysian pharmacists, though better than the Maltese, was lower than the scores obtained by psychologists and medical professionals reported in other study, with mean ADKS scores of 24.10 and 26.08, respectively (Smyth et al., 2013). The critical role and active participation of psychologists and medical professionals throughout the lifespan of the AD illness encompasses prevention, diagnosis, and treatment may attribute to their better knowledge on AD (American Psychological Association, 2014). It also appears that the pharmacists' working setting (i.e., hospital vs. health clinic) did not affect their total ADKS score.

It is interesting to note that pharmacists with a master education did not score better than the bachelor holders. It is unfortunately that the survey did not elicit the specialty of the master holders, and therefore, it is unclear whether AD was part of the learning focus during the master training. Of note, there was no difference in the mean ADKS score between the hospital pharmacists and health clinics pharmacists except in one domain (i.e., care giving). The score for the care giving domain was higher among the pharmacists in public hospitals compared to those worked in the health clinics. This could be due to the fact that hospital pharmacists have more encounters with AD patients or caregivers in the geriatric clinics or wards. The pharmacists scored lowest in domains such as symptom and course of disease. Indeed, these two domains have more medically-oriented questions, which may not be the primary focus of their job scopes, for example, the stages of cognitive impairment in people with AD and the lifespan of people with AD generally can survive.

There was a positive correlation between ADKS score and self-rated AD knowledge using a 11-point scale (*r* = 0.33, *p* < 0.001), consistent with the previous study which compared the AD knowledge among the healthcare providers (i.e., nursing, medical, allied health, and support) (Smyth et al., 2013). The respondents who rated themselves having low knowledge tend to score lower on ADKS. This shows a good self-awareness among the pharmacists on their inadequate knowledge on AD and may serve as a motivation for them to acquire latest information on AD, particularly, if they are to be provided with the opportunity via continuous professional development (CPD) or refresher courses.

In the current study, the age group (i.e., age <30) was found to be a significant independent predictor of higher ADKS score. This is consistent with an Australian study that surveyed the nursing, medical, support, and allied health staff, which revealed that age bracket of <30 years old scored 1.0 point ADKS higher than other age groups (Smyth et al., 2013). This could be due to the updated pharmacotherapy syllabus related to dementia and AD taught during the undergraduate pharmacy. Interestingly, unlike this study, age and duration of working did not correlate well with increased knowledge (Zerafa and Scerri, 2016). In fact, this highlights the need of CPD or refresher courses on AD to equip all pharmacists, regardless of seniority, with the latest information on AD. Targeted information on the symptoms and course of disease of AD may be, in particularly, relevant as the pharmacists scored poorly on these areas.

Malay pharmacists had a significantly higher ADKS score and were an independent predictor associated with higher ADKS score in the current study. This interesting finding could be related to their exposure and personal experience in handling AD among their family members due to the greater prevalence of AD among in Malay ethnicity (Kua and Ko, 1995; Kadir et al., 1997; Tey et al., 2016). Unlike developed countries where residential care is provided to older people especially those with AD, certain Asian countries, such as Malaysia, still rely on family members on their daily living (Scerri, 2016). This indirectly creates a higher awareness on AD and stimulates the interest among the Malay pharmacists to enhance their understanding on AD. The variance in the current study was lower (4.4%) than the study by Smyth et al. (i.e., 14.0%) (Smyth et al., 2013). This could be due to the fact that only one population (i.e., pharmacists), instead of diverse groups or different healthcare settings, was surveyed.

There are several limitations in this study. Some respondents may have referred to any resources while answering this questionnaire. In addition, the current study did not evaluate the previous training and personal experience on AD (e.g., family member with AD), which could have an impact on the ADKS score. Furthermore, this study was conducted at only one region in Malaysia (i.e., Selangor), and thus, the findings from this study cannot be generalized to all pharmacists in Malaysia.

## Conclusion

In the present study, the majority of the pharmacists have moderate ADKS score. No difference in AD knowledge was noted among the pharmacists working in public hospitals and health clinics except in the care giving domain. AD-specific education training would have benefited these pharmacists in providing better care to AD patients.

## Author contributions

THMN assisted in data collection, data analysis/interpretation and write-up. CFN assisted in concept/design, critical revision and approval of the article. YHH, SMJ, MFO, and LCM assisted in critical revision and approval of the article.

### Conflict of interest statement

The authors declare that the research was conducted in the absence of any commercial or financial relationships that could be construed as a potential conflict of interest.
